# Distinct Bacterial Communities in Surficial Seafloor Sediments Following the 2010 Deepwater Horizon Blowout

**DOI:** 10.3389/fmicb.2016.01384

**Published:** 2016-09-13

**Authors:** Tingting Yang, Kelly Speare, Luke McKay, Barbara J. MacGregor, Samantha B. Joye, Andreas Teske

**Affiliations:** ^1^Department of Marine Sciences, University of North Carolina, Chapel HillNC, USA; ^2^Department of Marine Sciences, University of Georgia, AthensGA, USA

**Keywords:** Deepwater Horizon, marine sediment, MOSSFA, marine snow, bacterial populations, *Cycloclasticus*

## Abstract

A major fraction of the petroleum hydrocarbons discharged during the 2010 Macondo oil spill became associated with and sank to the seafloor as marine snow flocs. This sedimentation pulse induced the development of distinct bacterial communities. Between May 2010 and July 2011, full-length 16S rRNA gene clone libraries demonstrated bacterial community succession in oil-polluted sediment samples near the wellhead area. Libraries from early May 2010, before the sedimentation event, served as the baseline control. Freshly deposited oil-derived marine snow was collected on the surface of sediment cores in September 2010, and was characterized by abundantly detected members of the marine *Roseobacter* cluster within the *Alphaproteobacteria*. Samples collected in mid-October 2010 closest to the wellhead contained members of the sulfate-reducing, anaerobic bacterial families *Desulfobacteraceae* and *Desulfobulbaceae* within the *Deltaproteobacteria*, suggesting that the oil-derived sedimentation pulse triggered bacterial oxygen consumption and created patchy anaerobic microniches that favored sulfate-reducing bacteria. Phylotypes of the polycyclic aromatic hydrocarbon-degrading genus *Cycloclasticus*, previously found both in surface oil slicks and the deep hydrocarbon plume, were also found in oil-derived marine snow flocs sedimenting on the seafloor in September 2010, and in surficial sediments collected in October and November 2010, but not in any of the control samples. Due to the relative recalcitrance and stability of polycyclic aromatic compounds, *Cycloclasticus* represents the most persistent microbial marker of seafloor hydrocarbon deposition that we could identify in this dataset. The bacterial imprint of the DWH oil spill had diminished in late November 2010, when the bacterial communities in oil-impacted sediment samples collected near the Macondo wellhead began to resemble their pre-spill counterparts and spatial controls. Samples collected in summer of 2011 did not show a consistent bacterial community signature, suggesting that the bacterial community was no longer shaped by the DWH fallout of oil-derived marine snow, but instead by location-specific and seasonal factors.

## Introduction

The Deepwater Horizon (DWH) oil spill released more than 4.9 million barrels of crude oil into the Gulf of Mexico (McNutt et al., 2012). Available oil budgets indicated that in total about 25% of the spilled oil was recovered or skimmed and burned, between 28 and 34% were consumed via biological oxidation in deep waters (Joye et al., 2016), 2–15% sedimented to the seafloor (Valentine et al., 2014; Chanton et al., 2015), and the remaining 32–39% did not have a clear environmental fate (Joye et al., 2016). Large amounts of marine oil-derived snow formed at the sea surface within a few days of the wellhead explosion (Dell’Amore, 2010; Passow et al., 2012). As demonstrated in lab studies, these floating oil aggregates gradually lose their buoyancy due to increasing aggregation of phyto- and zooplankton debris, fecal pellets, oily particles and droplets into a sticky matrix of microbially produced extracellular polymeric substances (Passow et al., 2012; Passow, 2016). Oil uptake by zooplankton (Mitra et al., 2012) and subsequent packaging into fecal pellets may interact with aggregate formation, or shuttle petrocarbon to the seafloor directly (Muschenheim and Lee, 2002). In addition to particle-associated transport, the dynamic deepwater hydrocarbon plume, containing an estimated 50% of the discharged oil (Joye, 2015), is likely to have impacted the slope sediments of the northern Gulf of Mexico (reviewed in Daly et al., 2016). These modes of oil transport to the seafloor are effective, as shown by several lines of geochemical evidence: the chemical composition of the sedimented hydrocarbons was congruent with Macondo oil as its source (Mason et al., 2014); widespread sedimentation of large amounts of fossil petrocarbon, equivalent to approximately 3–5% of the total carbon emitted during the Deepwater Horizon blowout, was demonstrated by analysis of C-isotopic composition (δ^13^C and Δ^14^C) in seafloor hydrocarbon deposits (Chanton et al., 2015); sedimentation rates accelerated by two orders of magnitude during summer and early fall of 2010, as determined by analyzing the ^234^Th inventories of surficial sediment cores (Brooks et al., 2015); coinciding with the sedimentation pulse, redox conditions in the sediment changed, and showed biogeochemical signatures of microbial metal reduction in the upper sediments (Hastings et al., 2016).

The microbial communities in the surficial seafloor sediments respond and adapt to the availability of hydrocarbon-derived substrates, and gradual depletion of electron acceptors. Oil-impacted seafloor sediments (1.5–3 cm depth) from October 2010 harbored sulfate-reducing *Deltaproteobacteria* with genes indicating degradation of aromatic hydrocarbons (Kimes et al., 2013). Sediments collected in 2011 near the wellhead area have yielded sequence libraries suggesting members of the methylotrophic genera *Methylobacter* and *Methylococcus* and the phyla *Actinobacteria, Firmicutes*, and *Chloroflexi* as the most abundant groups (Liu and Liu, 2013). A large-scale metagenomic survey found that in sediments near the wellhead with peak concentrations of total petroleum hydrocarbons, microbial populations were structured by the concentrations of total petroleum hydrocarbons and bioavailable nitrogen (NH_3_ + NO_3_; Mason et al., 2014), suggesting links to anaerobic denitrification (supported by recovery of the functional genes of this pathway) but also to aerobic ammonia oxidation, the first step of nitrification.

Since these studies rely on sample sets collected at one time point or within short time frames, for example during a single cruise, we complement these investigations with a longterm timeline survey of bacterial sedimentary communities from May 2010 to July 2011, using nearly full length 16S rRNA gene clone libraries for maximum taxonomic specificity. We document the distinct occurrence patterns of oil-associated family- and genus-level bacterial groups over time, and suggest petrocarbon sedimentation and changing biogeochemical niches as factors that shape these bacterial communities in near-surface seafloor sediments.

## Materials and Methods

### Sampling

**FIGURE 1:**
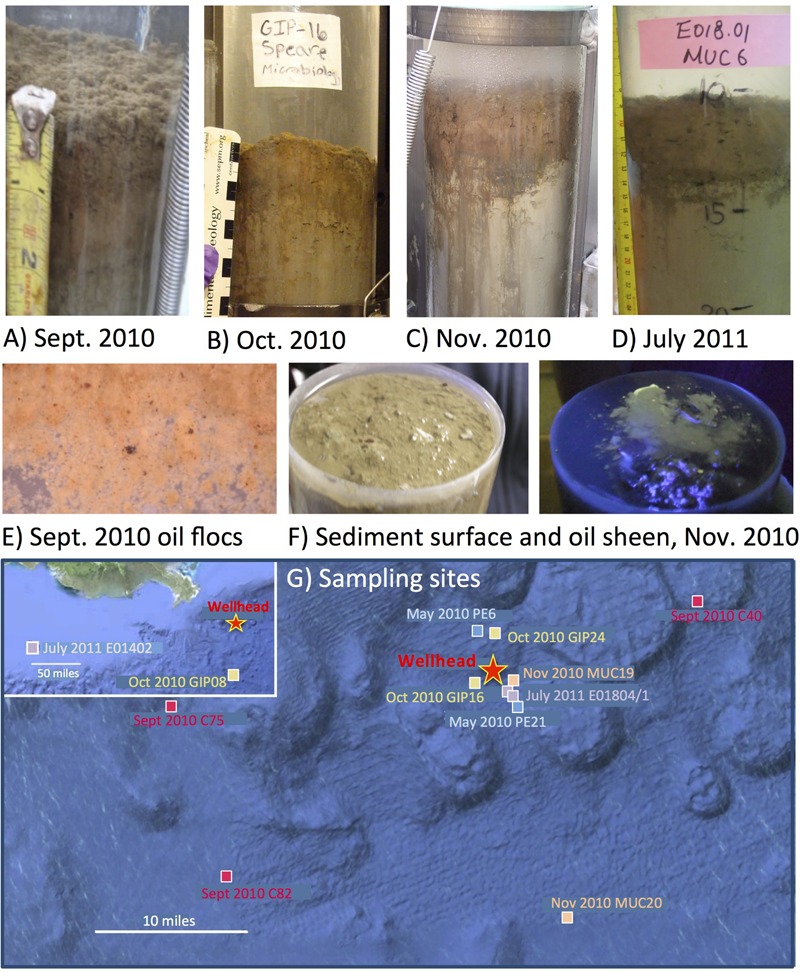
**Oil-contaminated sediment cores, characterized by a red-brown layer at the top of the gray sediment.** The photos show a representative core from the RV Oceanus cruise in September 2010 **(A)**, core GIP16 collected in Mid-October 2010 **(B)**, core MUC19 collected at the end of November 2010 **(C)**, and core E01801 collected in July 2011 **(D)**. Close-up of oil-derived marine snow flocs collected from the surface of 2010 September sediment, and spread in a Petri dish, with small dark oil droplets in millimeter size **(E)**. Surface of MUC 19 without and with UV illumination, showing the bare surface of the sediments, and the green fluorescence sheen under UV, indicating petroleum hydrocarbons **(F)**. Seafloor map of sampling sites around the Macondo wellhead **(G)**.

Our sediment samples were obtained from five different cruises (**Table 1**). The May 2010 samples were collected by boxcoring and subcoring on the first R/V *Pelican* oil spill cruise, 2 weeks after the spill occurred (Diercks et al., 2010a,b). These uniformly ochre-colored sediments from the wellhead area showed no visible oil floc deposition, had no hydrocarbon or sulfide smell, shared the low DIC concentrations (3–4 mM) of northern Gulf slope sediments (McKay et al., 2013), and lacked the redox-sensitive metal signatures of the oil-derived sedimentation pulse (Hastings et al., 2016). Thus, they provided controls for the seafloor conditions before the impact of oil-derived sedimentation. Subsequently, samples were collected by multicorer. In September 2010, oil-contaminated sediment cores were collected on R/V *Oceanus*; the sediment surface was now covered with floc-like deposits smelling noticeably of petroleum (**Figures 1A,E**), and fluorescing green under UV light. Seafloor sediments from the wellhead area with a conspicuous red-brown coloration of the upper 3–4 cm layer (**Figure 1B**), and well-documented concentrations of petroleum polyaromatic compounds (Woodruff, 2014), were collected in mid-October 2010 on R/V *Cape Hatteras*. Later sediment sample sets collected near the wellhead in late November 2010 with R/V *Atlantis* (Ziervogel et al., 2016b) as well as in July 2011 with R/V *Endeavor* retained the red-brown surface layer (**Figures 1C,D**), but lacked the hydrocarbon smell. The red-brown coloration represents precipitated MnO_2_ and ferric iron hydroxide; these metals were originally mobilized as reduced Fe^2+^ and Mn^2+^ after stimulation of microbial metal-reducing bacteria in oil-impacted sediment, and subsequently reoxidized and precipitated near the sediment surface as soon as oxidizing conditions returned; this redox signature was consistently absent in control cores (Hastings et al., 2016). All sediment cores were sectioned and stored immediately in -80°C freezers.

**Table 1:** Samples collected on multiple research cruises near the Macondo wellhead with sampling dates, depths, and geographical coordinates.

Sample names	Ship	Date	Depth (m)	Latitude (N)	Longitude (W)	Oil-impacted	Sample layer
PE6	RV *Pelican*	5/5/2010	1380	28°46.557	88°24.293	Negative	0–3 cm
PE21	RV *Pelican*	5/8/2010	1605	28°42.150	88°21.729	Negative	0–3 cm
C40	RV *Oceanus*	9/7/2010	1496	28°47.282	88°10.020	Oil flocs	0–3 cm
C75	RV *Oceanus*	9/10/2010	1087	28°42.650	88°44.900	Oil flocs	0–1 cm
C82	RV *Oceanus*	9/11/2010	1372	28°32.950	88°40.760	Oil flocs	0–1 cm
GIP24	RV *Cape Hatteras*	10/17/2010	1418	28°46.235	88°22.874	Surface brown layer	0–1 cm
GIP16	RV *Cape Hatteras*	10/16/2010	1560	28°43.383	88°24.577	Surface brown layer	0–1 cm
GIP16 (RNA)	RV *Cape Hatteras*	10/16/2010	1560	28°43.383	88°24.577	Surface brown layer	0–1 cm
GIP16 3–4 cm	RV *Cape Hatteras*	10/16/2010	1560	28°43.383	88°24.577	Below brown layer	3–4 cm
GIP08	RV *Cape Hatteras*	10/13/2010	2360	27°54.370	88°27.001	Negative	0–1 cm
GIP08 (RNA)	RV *Cape Hatteras*	10/13/2010	2360	27°54.370	88°27.001	Negative	0–1 cm
MUC19	RV *Atlantis*	11/30/2010	1574	28°43.350	88°21.770	Surface brown layer	0–2.5 cm
MUC20	RV *Atlantis*	12/1/2010	1885	28°29.290	88°19.050	Negative	0–2.5 cm
E01801	RV *Endeavor*	7/25/2011	1630	28°42.382	88°21.815	Surface brown layer	0–2 cm
E01804	RV *Endeavor*	7/26/2011	1620	28°42.491	88°21.999	Surface brown layer	0–2 cm
E01402	RV *Endeavor*	7/21/2011	64	28°20.919	91°49.563	Negative	0–2 cm

### DNA and RNA Extraction from Sediments

The surface layer from each sediment sample was used for DNA and RNA extraction (**Table 1**). Total sediment DNA was extracted from 0.25 g of each sample by using the PowerSoil^TM^ DNA isolation kit (MoBio Laboratories, Carlsbad, CA, USA), according to the manufacturer’s instruction manual. Sediment for RNA extraction was thawed in 4.5 M sodium trichloroacetic acid (TCA) lysis buffer (pH 7.0; McIlroy et al., 2008). The TCA lysis buffer and sample mixture was split between three baked shaking flasks that each contained 27 g 0.1 mm beads, 7 g 0.4 mm beads, 0.33 g polyvinylpolypyrrolidone, and 550 μL antifoam B. The contents of each flask were subjected to bead-beating in two intervals of 40 s at high speed in the bead beater machine (Miyatake et al., 2013). Samples were transferred to PPCO centrifuge tubes (Nalgene^TM^, Oak Ridge, TN, USA) and centrifuged for 20 min at 2500 rpm, 4°C. The supernatant was transferred to 50 mL Falcon^®^ tubes with a maximum of 30 mL per tube. Nucleic acids were precipitated in 0.6 volumes isopropanol overnight at -20°C, centrifuged for 30 min at 2500 rpm, 4°C, washed with 25 mL cold 70% ethanol, and then centrifuged again for 10 min at 2500 rpm, 4°C and air dried. Precipitated nucleic acid pellets were resuspended in 20 mL distilled nuclease-free water, split into two Teflon Oak Ridge centrifuge tubes, and extracted by multiple separations with low-pH (5.1) phenol, phenol–chloroform, and chloroform. For each extraction equal volume of either phenol, phenol–chloroform, or chloroform was added to the remaining aqueous RNA solution, vortexed for 30 s, and centrifuged for 10 min at 2500 rpm, 4°C. Following each extraction the aqueous layer was transferred to a new tube and the extraction was repeated until there was a clear interface. The final aqueous sample was transferred to a clean tube and precipitated overnight at -20°C in 0.7 by volume isopropanol and 0.5 by volume ammonium acetate (Lin and Stahl, 1995; MacGregor et al., 1997). Nucleic acid pellets were resuspended in 125 μL nuclease-free water, and purified with the RNeasy RNA cleanup kit (Qiagen, Germantown, MD, USA). One or more DNase treatments, using Turbo DNase I (Thermo Fisher Scientific, Waltham, MA, USA) either on the column during RNeasy cleanup or in solution or both, were necessary to eliminate PCR-detectable DNA.

### PCR Amplification and Cloning

The resuspended rRNA was reverse transcribed to cDNA first by the SuperScript^®^ III One-Step RT-PCR system with Platinum^®^ Taq DNA Polymerase (Thermo Fisher Scientific, Waltham, MA, USA) according to the recommended reaction conditions. Then the cDNA and the extracted total DNA were amplified with Speedstar DNA polymerase (TaKaRa, Shiga, Japan) using the bacterial primers 8f and 1492r (Teske et al., 2002) and the manufacturer’s recommended concentration for buffer, dNTPs and DNA polymerase. Each PCR reaction consisted of 2 μl DNA extract, 2.5 μl 10x FBI buffer (TaKaRa, Shiga, Japan), 2.0 μl dNTP mix, 2.0 μl 10 μM solution of primers 8F and 1492R, respectively (both primers from Invitrogen, Carlsbad, CA, USA), and 0.25 μl SpeedStar polymerase (TaKaRa), and was brought to 25 μl with sterile H_2_O. Amplification was performed in a BioRad iCycler Thermal Cycler (BioRad, Hercules, CA, USA) as follows: initial denaturation at 95°C for 4 min, 25 cycles of 95°C (10 s), 55°C (15 s) and 72°C (20 s), and a final 10 min extension of 72°C. PCR and RT-PCR product aliquots, including positive and negative controls, were SYBR green stained and visualized using a 1.5% agarose gel. The products were purified using the MinElute^®^ PCR Purification Kit according to the manufacturer’s instructions (Qiagen, Valencia, CA, USA), and cloned into OneShot^®^ TOP10 competent Cells using the TOPO TA Cloning^®^ Kit for Sequencing (Invitrogen, Carlsbad, CA, USA) according to the manufacturer’s instructions. Transformed cells were grown on LB/Xgal/Kanamycin plates. Individual white colonies were arbitrarily picked, re-plated and sanger-sequenced at Genewiz Corporation (South Plainfield, NJ, USA) using vector primers M13 F and M13 R.

### Phylogenetic Analysis

Near-complete 16S rRNA gene sequences were analyzed using Sequencher (Gene Codes, Ann Arbor, MI, USA) and compared to other sequences via the Basic Local Alignment Search Tool (BLAST) of the National Center for Biotechnology Information^1^ (Altschul et al., 1990). The sequences were examined for chimeras using Pintail 1.1 software (Ashelford et al., 2005). After construction of a general 16S rRNA alignment using the ARB phylogeny software package (Ludwig et al., 2004) and the SILVA 115 database (Pruesse et al., 2007), separate alignments for the *Gamma*- and *Alphaproteobacteria* were prepared with sequences for related *Gammaproteobacteria* and *Alphaproteobacteria*. Sequences of well-characterized pure cultures and described species were used for phylogenies whenever possible; otherwise, molecular phylotypes with an informative literature history were selected to anchor major phylogenetic branches of uncultured bacteria. The identifications were made based on a full-length, manually curated ARB alignment for all 2200 sequences. Phylogenetic trees were constructed and bootstrap checks (1000 reruns) of the tree topology were performed using ARB’s neighbor-joining function with Jukes-Cantor correction.

### Statistical Analysis

To compare the homogeneity of the sediment 16S rRNA and rDNA clone libraries, principal coordinates analysis (PCoA) hierarchical clustering was performed by using UniFrac online program http://bmf.colorado.edu/unifrac/ (Lozupone et al., 2011) using a NJ tree generated with ARB software. X-Fig^2^ was used to edit image files obtained from the ARB software when necessary.

### Database Access

The 16S rRNA gene sequences were submitted to GenBank and are accessible under GenBank Numbers KX172179–KX173285. Specifically, the GenBank numbers for sample C40 are KX172179–KX172328; sample C75, KX172329–KX172396; sample C82, KX172397–KX172514; sample E014, KX172515–KX172558; sample E01801, KX172559–KX172596; sample E01804, KX172597–KX172667; sample GIP08, KX172668–KX172718; sample GIP08RNA, KX172719–KX172771; sample GIP16-0-1 cm, KX172772–KX172892; sample GIP16-3-4 cm, KX172893–KX172944; sample GIP16RNA, KX172945–KX172993; sample GIP24, KX172994–KX173048; sample MUC19, KX173049–KX173105; sample MUC20, KX173106–KX173165, sample PE6, KX173166–KX173236; sample PE21, KX173237–KX173285.

## Results

### Bacterial Community Change at Phylum or Class Resolution

**FIGURE 2:**
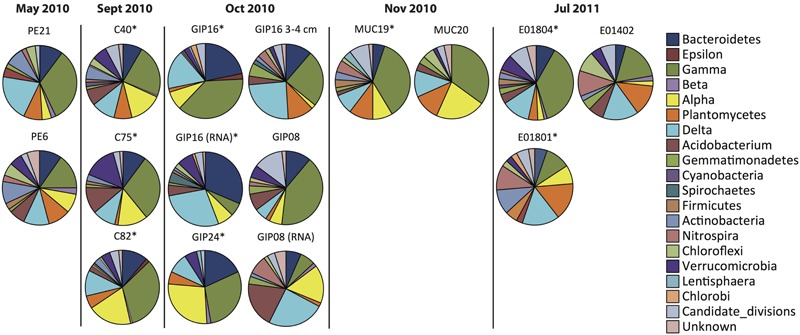
**Phylum-level resolution pie chart plots of bacterial 16S rRNA gene clone libraries and reverse-transcript 16S rRNA clone libraries from oil polluted sediment samples (labeled with ^∗^) and control sites**.

The earliest sediment samples of this dataset were collected during the first oil spill cruise on R/V *Pelican* on May 5 and 8, 2010, during the third week after the onset of the DWH blowout (**Table 1**). Sediment cores PE6 and PE21 were collected 3 miles northwest and 2.7 miles southeast of the wellhead, respectively (**Figure 1**). Neither hydrocarbon smell nor oily flocs were found on these sediments; cores collected on the same cruise that were examined for geochemical signatures of oil floc-derived sedimentation (including core PE6) also lacked the geochemical signature of hydrocarbon-induced microbial metal mobilization that characterizes oil-impacted sediments (Hastings et al., 2016). Therefore, these sediment samples are control sediments near the wellhead that represent the microbial community baseline of surficial sediments before oil deposition. Five bacterial phyla or sub-phyla were abundant in the bacterial 16S rRNA gene clone libraries of cores PE6 and PE21: *Gammaproteobacteria* (15.5, 32.7%), *Deltaproteobacteria* (11.3, 20.4%), *Bacteroidetes* (9.9, 10.2%), *Planctomycetes* (9.9, 8.2%), and *Actinobacteria* (9.9, 8.2%; **Figure 2**; **Table 2**). The PE6 and PE21 clone libraries also shared the minority groups *Betaproteobacteria, Alphaproteobacteria, Acidobacteria, Gemmatimonadetes, Nitrospira*, and *Chloroflexi*. Members of the phyla *Verrucomicrobia* and *Firmicutes* were only found in PE6 but not in PE21. In general, PE6 and PE21 shared the major bacterial groups of control surface sediment in a previous Gulf of Mexico bacterial community survey (Lloyd et al., 2010).

**Table 2:** Percentage of main phyla in 16 sediment bacterial 16S rRNA and rRNA gene clone libraries from May 2010 to July 2011.

	May 2010 non-oily		September 2010 oily			October 2010 oily			October 2010 non-oily			November 2010 oily	November 2010 non-oily	July 2011 oily		July 2011 non-oily
	PE6	PE21	C40	C75	C82	GIP24	GIP16	GIP16 (RNA)	GIP163–4 cm	GIP08	GIP08 (RNA)	MUC19	MUC20	E01801	E01804	E014
*Bacteroidetes*	9.9%	10.2%	8.6%	10.3%	11.8%	18.2%	22.1%	32.5%	11.5%	11.8%	7.3%	5.3%	0.0%	5.3%	8.5%	4.5%
Epsilon	0.0%	0.0%	0.0%	0.0%	1.7%	0.0%	2.5%	0.0%	0.0%	0.0%	0.0%	0.0%	0.0%	0.0%	0.0%	0.0%
Gamma	15.5%	32.7%	23.2%	29.4%	32.8%	29.1%	37.7%	6.0%	25.0%	43.1%	7.3%	36.8%	35.0%	10.5%	38.0%	18.2%
Beta	2.8%	2.0%	0.7%	0.0%	0.8%	1.8%	0.0%	0.0%	0.0%	0.0%	1.8%	0.0%	0.0%	0.0%	1.4%	2.3%
Alpha	8.5%	4.1%	14.6%	13.2%	19.3%	27.3%	7.4%	7.2%	1.9%	5.9%	18.2%	8.8%	21.7%	7.9%	2.8%	2.3%
*Plantomycetes*	9.9%	8.2%	7.9%	1.5%	5.9%	5.5%	1.6%	0.0%	11.5%	2.0%	1.8%	8.8%	11.7%	15.8%	2.8%	11.4%
Delta	11.3%	20.4%	4.6%	4.4%	8.4%	9.1%	17.2%	28.9%	23.1%	5.9%	21.8%	8.8%	11.7%	15.8%	12.7%	15.9%
*Acidobacterium*	7.0%	4.1%	7.3%	11.8%	2.5%	0.0%	0.8%	4.8%	3.8%	7.8%	21.8%	1.8%	3.3%	2.6%	4.2%	6.8%
*Gemmatimonadetes*	1.4%	2.0%	1.3%	1.5%	0.8%	0.0%	0.0%	1.2%	5.8%	3.9%	3.6%	1.8%	3.3%	0.0%	2.8%	4.5%
*Cyanobacteria*	0.0%	0.0%	2.0%	0.0%	0.8%	0.0%	0.0%	0.0%	0.0%	0.0%	0.0%	0.0%	0.0%	0.0%	1.4%	0.0%
*Spirochaetes*	0.0%	0.0%	0.0%	0.0%	0.0%	0.0%	0.8%	4.8%	1.9%	0.0%	0.0%	0.0%	0.0%	0.0%	0.0%	0.0%
*Firmicutes*	2.8%	0.0%	1.3%	1.5%	0.0%	0.0%	0.0%	1.2%	0.0%	0.0%	0.0%	3.5%	0.0%	5.3%	2.8%	0.0%
*Actinobacteria*	9.9%	8.2%	6.0%	2.9%	3.4%	0.0%	0.0%	1.2%	1.9%	0.0%	1.8%	3.5%	0.0%	10.5%	2.8%	2.3%
*Nitrospira*	2.8%	2.0%	0.7%	0.0%	0.0%	0.0%	0.0%	0.0%	3.8%	2.0%	9.1%	5.3%	0.0%	10.5%	1.4%	11.4%
*Chloroflexi*	5.6%	4.1%	3.3%	0.0%	0.8%	0.0%	0.0%	2.4%	1.9%	2.0%	0.0%	1.8%	5.0%	2.6%	0.0%	9.1%
*Verrucomicrobia*	5.6%	0.0%	6.6%	14.7%	4.2%	5.5%	2.5%	6.0%	1.9%	3.9%	0.0%	0.0%	1.7%	2.6%	7.0%	4.5%
*Lentisphaera*	0.0%	0.0%	0.0%	0.0%	0.0%	1.8%	0.8%	0.0%	0.0%	0.0%	0.0%	3.5%	0.0%	0.0%	0.0%	0.0%
*Chlorobi*	0.0%	0.0%	0.0%	0.0%	0.0%	0.0%	2.5%	1.2%	0.0%	0.0%	0.0%	0.0%	0.0%	2.6%	0.0%	0.0%
Candidate_divisions	2.8%	2.0%	10.6%	8.8%	5.0%	1.8%	4.1%	2.4%	3.8%	11.8%	3.6%	8.8%	3.3%	5.3%	7.0%	6.8%
Unknown	4.2%	0.0%	1.3%	0.0%	1.7%	0.0%	0.0%	0.0%	1.9%	0.0%	1.8%	1.8%	3.3%	2.6%	4.2%	0.0%

**FIGURE 3:**
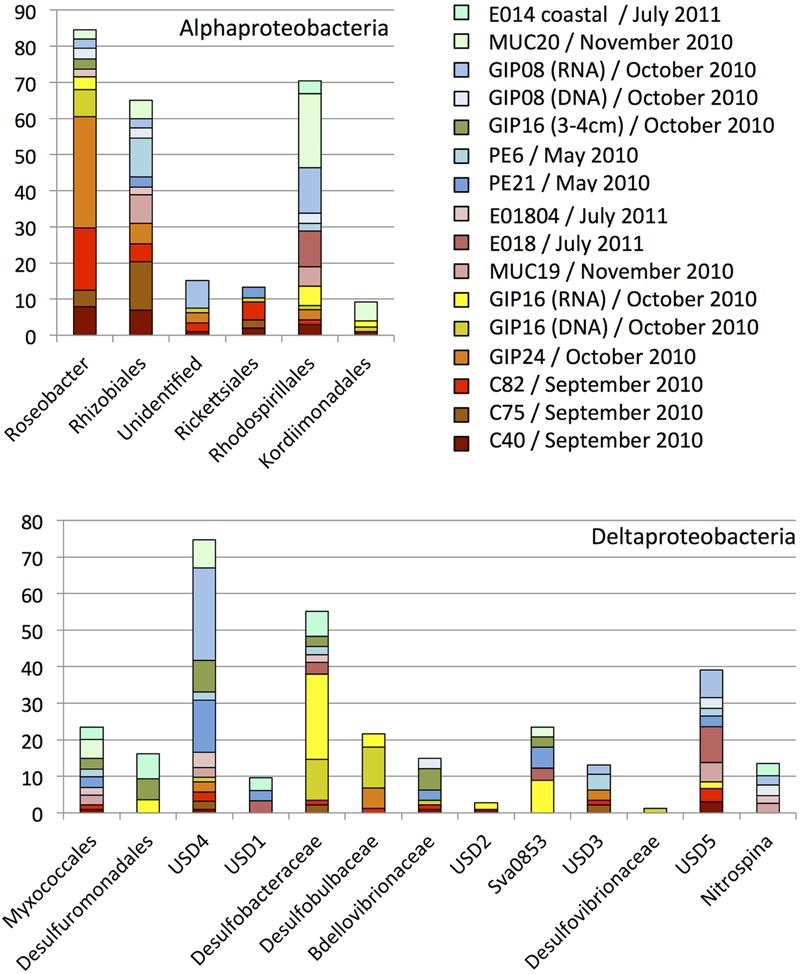
**Order, family- and genus-level identification of 16S rRNA clones representing the *Alphaproteobacteria* and *Deltaproteobacteria* in the sequence dataset shown in **Figure 2** at phylum-level resolution.** The y-axis shows the total number of sequences. The color key identifies the 16S rRNA gene contributions for every sediment sample. Yellow, orange, red and brown colors were chosen for oil-contaminated samples, and green and blue colors were chosen for control samples lacking visible oil impact.

Recovered during the September 2010 R/V *Oceanus* cruise, the first oil-impacted sediment cores of this sample set were characterized by a brown, flocculent layer of marine snow-like deposits overlaying ochre-colored seafloor sediment. This overlying material contained small droplets and particles of weathered oil (**Figure 1E**) similar to oil droplets observed embedded in freshly formed, sinking marine snow (Passow et al., 2012); the samples were also characterized by strong petroleum smell. Three samples of oil-contaminated, recently deposited marine snow flocs from surficial sediments (C40, C75, and C82) were examined, from sampling sites 14 miles northeast, 21 miles west, and 22 miles southwest of the wellhead (**Figure 1**). While the *Gammaproteobacteria* dominated in the 16S rRNA gene clone libraries C40, C75, and C82 (23.2, 29.4, and 32.8%), the *Alphaproteobacteria* had become the second-most abundant group in all three samples (14.6, 13.2, 19.3%; **Figures 2** and **3**). This change is consistent with the alphaproteobacterial bloom detected in independent metagenomic analyses of seafloor sediments collected in September 2010 (Mason et al., 2014). *Deltaproteobacteria* and *Bacteroidetes* were still within the most frequently detected groups, but the *Actinobacteria* decreased to a similar level as other minority groups. The *Planctomycetes* contributed significantly to clone libraries of C40 and C82 (7.9 and 5.9%), but were barely found in C75 (1.5%; **Table 2**).

One month later, more oil-contaminated cores were recovered from the wellhead area during the October 2010 R/V *Cape Hatteras* cruise. At sites GIP16 (1.6 miles west-southwest to the wellhead), and GIP24 (2.4 miles north-northeast of the wellhead), the reddish surface sediment layers with UV-fluorescent oil spots and petroleum odor were recovered again (**Figure 1**). *Gammaproteobacteria* (37.7, 29.1%) contributed most abundantly to the 16S rRNA gene clone libraries of both GIP16 and GIP24; the contribution of the *Bacteroidetes* (22.1, 18.2%) almost doubled in October compared to the September samples (**Figure 2**). Interestingly, *Deltaproteobacteria* increased sharply (17.2%) while *Alphaproteobacteria* decreased (7.4%) in GIP16, whereas in GIP24 both groups persisted at similar levels (9.1% *Deltaproteobacteria*, 27.3% *Alphaproteobacteria*) as in September. The remaining minority bacterial groups in total accounted for 15.6 and 16.4% in both GIP samples.

Since the oil-derived sedimentation pulse covered the pre-spill sediment surface but did not appear to be mixed in, the pre-spill sediment microbial community could have remained intact in the underlying sediment. To check this possibility, the 3–4 cm sediment layer of core GIP16, right below the red-brown colored surface sediment, was sampled and sequenced. The 16S rRNA gene clone library results showed that this sample diverged from the immediately overlying surface sediment, but it resembled the pre-spill sediment samples PE6 and PE21 collected in May 2010. The 3–4 cm layer and the surficial sediment of GIP16 shared nine microbial groups, but 13 groups on phylum and sub-phylum level were shared between GIP16 3–4 cm, PE6, and PE21 (**Table 2**). These three samples also shared a low proportion of *Bacteroidetes* and a high percentage of *Planctomycetes*, in contrast to the GIP16 surface sample.

A spatial reference core (GIP08) without visible hydrocarbon pollution was taken 57 miles south of the wellhead (**Figure 1**). Its clone library demonstrated a distinct bacterial community dominated by the highest proportion of *Gammaproteobacteria* (43.1%) and of Candidate Divisions OD1/OP11 (11.8%) compared to all other samples. The *Bacteroidetes* accounted for 11.8% (**Figure 2**; **Table 2**). The GIP08 and GIP16 3–4 cm libraries shared 12 groups, seven of which were found at less than 8% each. The *Planctomycetes* and *Deltaproteobacteria* were found in lower percentages (2.0 and 5.9%) in GIP08, compared to 11.5 and 23.1% in GIP16 3–4 cm.

To identify active bacterial groups with increased rRNA content, we compared the reverse-transcribed 16S rRNA clone libraries of the oil-impacted core GIP16 and the reference core GIP08 to each other and to their corresponding 16S rRNA gene clone libraries. The two sediments showed distinct active phylum-level groups. In the GIP16 clone library of 16S rRNA transcripts, the *Bacteroidetes* (32.5%) and *Deltaproteobacteria* (28.9%) were the two most abundant groups; the remaining 11 groups accounted for less than 8% each (**Figure 2**). In contrast, in the 16S rRNA clone library of GIP08, *Deltaproteobacteria* (21.8%), *Acidobacteria* (21.8%) and *Alphaproteobacteria* (18.2%) predominated, while other groups contributed below 10%. The *Gammaproteobacteria*, the dominant group in DNA-based sediment clone libraries of these samples, contributed much less in the reverse-transcribed 16S rRNA gene clone libraries (6.0 and 7.3% in GIP16 and GIP08, respectively), suggesting that the *Gammaproteobacteria* contributed less to overall cellular activity and rRNA gene expression than to DNA presence.

The wellhead area was revisited and resampled in late November 2010 with R/V *Atlantis* and submersible *Alvin* (**Table 1**). The sediment core MUC19 was recovered from approximately 1.7 miles southeast of the wellhead (**Figure 1**); it showed a red-brownish surface layer about 4.5 cm thick but lacked noticeable petroleum smell (Ziervogel et al., 2016b). Under UV light, MUC19 showed fluorescent spots suggesting oil in the supernatant water (**Figure 1**), a possible consequence of residual leakage of fresh hydrocarbons around the DWH site. A reference core MUC20 was taken 17.4 miles south-southeast of the wellhead; the entire core was evenly ochre-gray colored, and lacked the red–brown surface layer. These sediments showed similar total organic carbon and total nitrogen content, microbial cell numbers and enzymatic activities (Ziervogel et al., 2016b). While the *Gammaproteobacteria* (36.8%) represented the most abundant clone library group of MUC19, the proportion of *Planctomycetes* increased compared to the September and October 2010 surface samples, and these sequences became the second-most dominant clone library group (8.8%). All other groups accounted for less than 10% individually. In MUC20, members of the *Gammaproteobacteria* (35%), *Alphaproteobacteria* (21.7%), *Planctomycetes* (11.7%), and *Deltaproteobacteria* (11.7%) in total accounted for more than 80% of the clone library (**Figure 2**; **Table 2**).

In July 2011, 1 year after the wellhead was capped, two cores E01801 and E01804 were taken 0.26 miles from each other, ca. 2 miles south-east to the DWH site, close to the May 2010 sampling site of core PE21 (**Figure 1**). The cores showed the red-brown surface layer known from the 2010 oil-impacted cores. Members of the *Planctomycetes*, especially of the *Phycisphaera mikurensis* group, dominated the clone library (15.8%, the *P. mikurensis* group counts for 10.5%) in the surface sediment of core E01801; the *Deltaproteobacteria* were found in the same proportion (15.8%), followed by the *Gammaproteobacteria, Actinobacteria*, and *Nitrospira* (10.5% each), and the other nine minority groups (<8% each; **Figure 2**). In the replicate core E01804, the *Planctomycetes* accounted for only 2.8% and represented one of the 13 microbial groups that were represented in smaller proportions, whereas *Gammaproteobacteria* (38.0%) and *Deltaproteobacteria* (12.7%) dominated the library, as previously seen in PE21. Such strongly divergent bacterial communities in cores from the same marine area suggest patchily distributed seafloor communities, possibly impacted by episodic particle resuspension at the sediment/seawater interface (Ziervogel et al., 2016a). The *Gammaproteobacteria, Deltaproteobacteria*, and *Planctomycetes* appeared again as the most frequently detected phyla in the shallow-water reference core E01402 (depth 64 m) 211 miles west of the wellhead, with higher proportions of *Nitrospira* and *Chloroflexi* than in deepwater cores (**Figure 2**).

### Bacterial Community Changes at Order, Family, and Genus-Level Resolution

In general, *Alphaproteobacteria, Deltaproteobacteria, Gamma-proteobacteria, Bacteroidetes, Planctomycetes*, and *Verrucomi crobia* occurred in high proportions in most sediment clone libraries, but their role in oil degradation remained hard to infer since the classification at phylum or sub-phylum level is not precise enough for functional or ecological inferences. Therefore, we fine-tuned our results by further analyzing the 16S rRNA sequences at the level of order, family or genus of these dominant groups.

### Alphaproteobacteria

The *Alphaproteobacteria* in the sediments represented six family- or order-level subgroups (**Figure 3**), mostly the monophyletic marine *Roseobacter* clade. This clade is physiologically highly diversified and includes genera and species capable of aerobic or nitrate-reducing heterotrophic degradation of dissolved and particular organic substrates, oxidation of inorganic and organic sulfur compounds (Lenk et al., 2012) or carbon monoxide as auxiliary electron and energy sources, anoxygenic photosynthetic sulfur oxidation, or aromatic hydrocarbon degradation (Wagner-Döbler and Biebl, 2006). *Roseobacter* genome studies revealed its potential for numerous biogeochemically relevant activities, including carbon monoxide oxidation, sulfur oxidation, dimethylsulfoniopropionate demethylation, aromatic compound degradation, denitrification, and phosphonate utilization (Buchan and González, 2010). While the marine *Roseobacter* clade was not found in sediment clone libraries before the oil fallout had settled on the seafloor (PE6 and PE21), it dominated the *Alphaproteobacteria* in the 2010 September samples C40, C75, and C82. The *Roseobacter* clones remained conspicuous in the October sample GIP24, where they contributed 20% of the clone library (the GIP24 *Roseobacter* accounted for 36% of the total *Roseobacter* sequences derived from all samples). The *Roseobacter* group was still detectable in GIP16 at lower abundance (9% of total *Roseobacter* sequences). Reference sediments without visible oil-derived sedimentation and red-brown coloration yielded *Roseobacter* sequences only in small numbers or not at all. These results suggest that *Roseobacter* are enriched in oil-derived marine aggregates, likely in response to the availability of substituted aromatic compounds, polysaccharides and other high-molecular weight substrates derived from decaying algae, as in a typical late-stage algal bloom (Dang and Lovell, 2016). Considering that members of the *Roseobacter* clade were detected frequently in laboratory-generated marine flocs growing on weathered Macondo crude oil collected from the sea surface (Arnosti et al., 2016), enrichment, transport and seafloor accumulation of *Roseobacter* clade members in marine oil snow is plausible.

Several studies suggested that members of the marine *Roseobacter* cluster play an important role in alkane and PAH degradation. Culture-independent studies suggested that members of the marine *Roseobacter* cluster were enriched in decane, hexadecane and other alkane-degrading seawater microcosms, but may be inhibited by some components of crude oil (McKew et al., 2007). Cultured representatives of the *Roseobacter* cluster were isolated from mangrove sediment and seawater by using media amended with pyrene, naphthalene, fluoranthrene, or phenanthrene (Brito et al., 2006; Pinyakong et al., 2012). Aromatics degradation pathways were identified in several *Roseobacter* cluster genomes (Buchan and González, 2010), including in a recently described new species and genus, *Tritonibacter horizontis*, from DWH surface oil flocs that can utilize substituted aromatics as sole carbon source (Klotz, 2016). The decline of *Roseobacter* cluster phylotypes in GIP16 and after October 2010 could be linked to increasing substrate limitation, as the available substrates transitioned from short hydrocarbons to more recalcitrant ones such as PAHs. Also, the cold *in situ* temperatures on the seafloor of deep Gulf of Mexico slope (ca. 4–5°C; Yang et al., 2016) may inhibit the physiological activity of surface-derived *Roseobacter* clade populations and select against them. While *Roseobacter* populations native to cool environments persist in oil enrichment studies at 4°C (Coulon et al., 2007), the *Roseobacter* clade isolate *Tritonibacter horizontis* from surface oil slicks has an optimal growth temperature of 30°C, close to the sea surface temperature of 26°C at the time of sampling, and a growth temperature range of 4–45°C (Klotz, 2016). In consequence, cold-adapted sediment bacteria with otherwise similar growth requirements and ecophysiological preferences for particle attachment – for instance, members of the *Bacteroidetes* that were detected frequently in the October samples – could outcompete the warm-water *Roseobacter* clade arrivals from the sea surface (Dang and Lovell, 2016).

### Deltaproteobacteria

**FIGURE 4:**
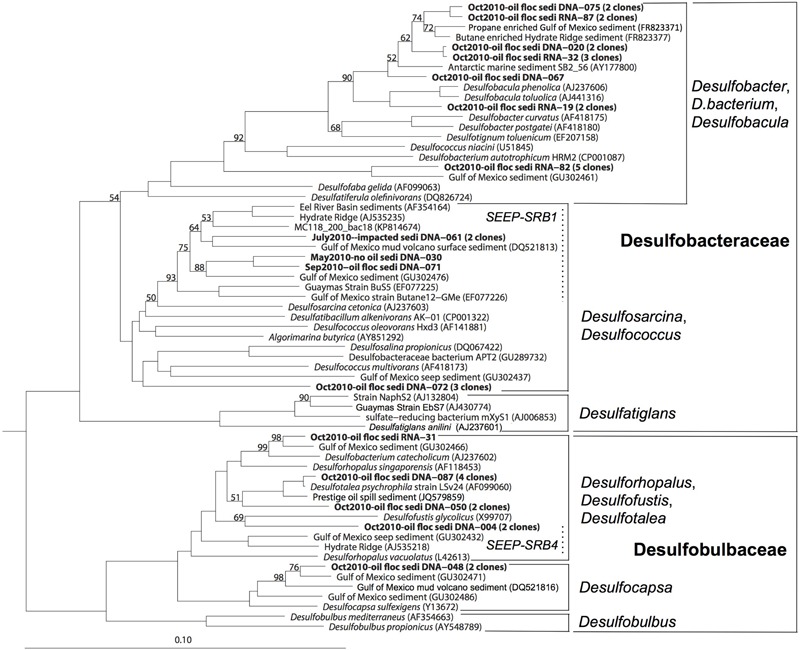
**16S rRNA gene phylogenetic tree of the deltaproteobacterial families *Desulfobacteraceae* and *Desulfobulbaceae*.** The scale bar corresponds to 10% sequence distance (Jukes-Cantor). The phylogeny was rooted with the Gammaproteobacterium *Colwellia psychrerythraea*.

The numerous natural hydrocarbon seeps in the Gulf of Mexico provide suitable permanent habitats for sulfate-reducing hydrocarbon-oxidizing bacteria (Lloyd et al., 2006, 2010; Teske, 2010; Kleindienst et al., 2012; Ruff et al., 2015). Here, members of the sulfate-reducing bacterial (SRB) family *Desulfobacteraceae* including the *Desulfosarcina*/*Desulfococcus* (DSS) clade, the *Desulfobulbaceae*, and two uncultured clusters (called here “uncultured sediment dwellers,” USD4 and USD5) constituted the principal deltaproteobacterial groups in these sediment samples (**Figure 3**). Some members of the DSS group can degrade short chain alkanes under strict anoxic conditions in coastal as well as deep seafloor sediments (Kniemeyer et al., 2007; Jaekel et al., 2013; Kleindienst et al., 2014). The *Desulfobacteraceae* and *Desulfobulbaceae* clades included sequences from the oil-polluted surficial sediments GIP24 and GIP16 (both 16S rRNA gene sequences and the reverse-transcript 16S rRNA cDNA sequences), and few sequences from non-polluted surface sediments (**Figure 4**).

Members of *Desulfobacteraceae* and *Desulfobulbaceae* were previously identified at natural seeps, mud volcanoes, and gas hydrates in the Gulf of Mexico (Lloyd et al., 2010; Orcutt et al., 2010), but also from the oil spills, such as the Prestige oil spill on the coast of northern Spain (Acosta-Gonzalez et al., 2013). In this case, the evidence linked the clones in GIP16 and GIP24 to the Deepwater Horizon oil spill, not to natural seepage: the GIP16 and GIP24 cores lacked methane gas pockets, gas hydrate or porewater sulfide accumulation. In contrast to seep sediments (Lloyd et al., 2006, 2010), the *Desulfobacteraceae* and *Desulfobulbaceae* clones were limited to the surface sediment layers, except for a single *Desulfobacteraceae* clone from the 3–4 cm layer of the GIP16 core. Secondly, the surficial fluffy sediment showed much smaller grain sizes than the sediments below this layer, indicating a specific depositional event (Brooks et al., 2015). Furthermore, isotopic analysis using Th234 as a short-lived marker (half-life time 24 days) for fast sedimentation pulses showed this layer was deposited within a few months, which is congruent with the timeline of the oil spill (Brooks et al., 2015; Chanton et al., 2015). Finally, the phylogenetic analysis of SRB groups demonstrated that the majority of the SRB sequences from October 2010 formed distinct clades that differed from the SRB clades (SEEP-SRB1 to SEEP-SRB4; Knittel et al., 2003) that are frequently found in natural cold seeps. Most phylotypes formed sister lineages to cultured aromatics-degrading *Desulfobacula* and alkane enrichments within the Desulfobacteraceae, or the psychrophilic genus *Desulfotalea* within the *Desulfobulbaceae*; phylogenetic overlap of the October sediment clones with potentially seep-dwelling SRB was limited to the SEEP-SRB-1 clade (**Figure 4**).

The *Desulfobacteraceae* and *Desulfobacteraceae* are anaerobes, and oxic surface sediments are by no means their favorite natural habitat. The appearance of these obligate anaerobes indicates changing, increasingly reducing redox conditions within the surficial oily sediment, a conclusion supported by the proliferation of nitrate-reducing genes detected in sediment metagenomes (Mason et al., 2014), and by the geochemical imprint of microbial metal reduction and mobilization pulses in oil-impacted surficial sediments (Hastings et al., 2016). Possibly, anoxic and reducing conditions that favor the development of SRB in surficial sediment result from oxygen consumption by *Roseobacter* clade members and *Bacteroidetes* that grow on suitable hydrocarbons and organic matter of planktonic origin as carbon source. Following this scenario, heterotrophic activity could sharply lower the oxygen concentration toward the center of the oil aggregates, and generate micro-scale anoxic niches. The anaerobic SRBs could then thrive in these anoxic particles and degrade remaining recalcitrant high-molecular weight aromatic hydrocarbons. Other deltaproteobacterial subgroups, such as the *Myxococcales* which accumulated in oil polluted sediment after the Prestige oil spill (Acosta-Gonzalez et al., 2013), were not detected forming temporary blooms in the Gulf of Mexico sediment.

### Gammaproteobacteria

**FIGURE 5:**
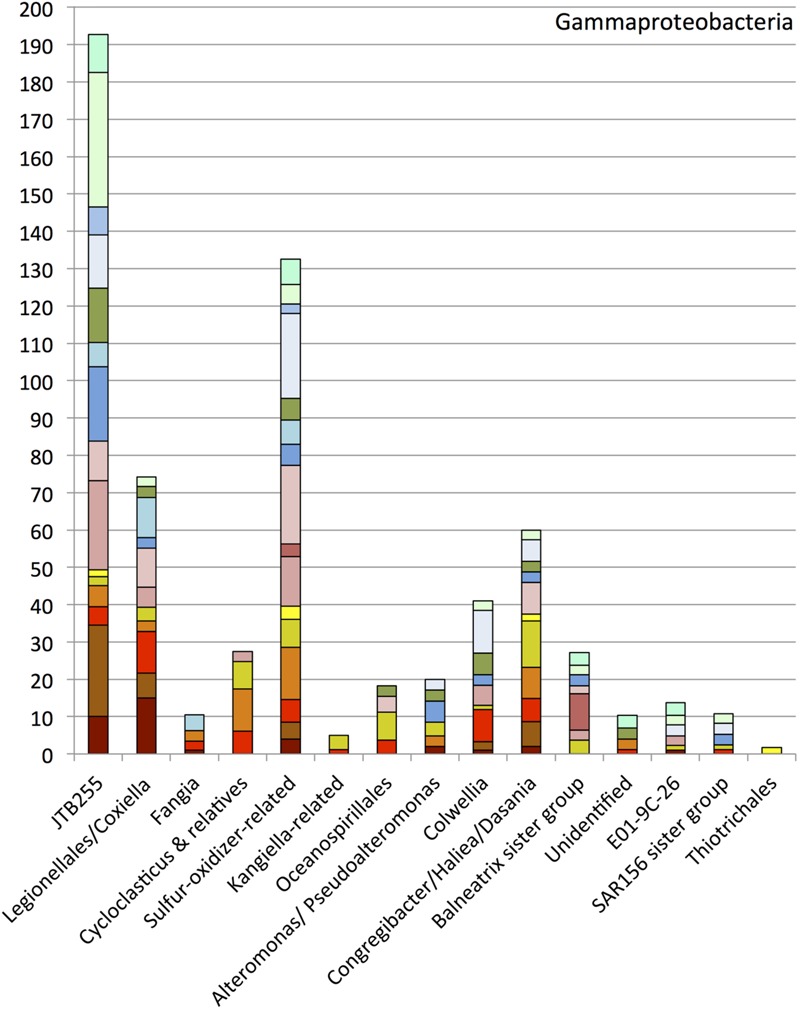
**Order, family- and genus-level identification of 16S rRNA clones representing the *Gammaproteobacteria* in the sequence dataset shown in **Figure 2** at phylum-level resolution**.

Members of the *Gammaproteobacteria* constituting 15 subgroups were found in high proportions in all clone libraries except the sample E01801 from July 2011 (**Figure 5**). The most abundant group, designated JTB255, was previously found in deep cold seep sediments of the Japan Trench (Li et al., 1999), and in diverse marine seafloor environments (Teske et al., 2011). Oil input apparently did not influence the occurrence pattern of the JTB255 group (**Figure 5**). A polyphyletic assemblage of sulfur-oxidizer-related bacteria, and a cluster including members of the *Legionellales, Coxiella* and *Rickettsiella*, constituted the next-most abundant gammaproteobacterial groups after JTB255. Sequences of the *Oceanospirillales* were found in small numbers in the sediments, but they did not fall into the DWH *Oceanospirillales* group recovered from the hydrocarbon plume (Yang et al., 2016). The remaining minor groups include the genera *Congregibacter*/*Haliea*/*Dasania, Fangia*, a *Kangiella*-related group, *Alteromonas*/*Pseudoalteromonas*, a *Balneatrix* sister group, the E01-9C-26 group, a SAR156 sister group and the *Thiotrichales*. The genera *Cycloclasticus* and *Colwellia* accounted for small portions of the gammaproteobacterial phylotypes.

### Cycloclasticus

**FIGURE 6:**
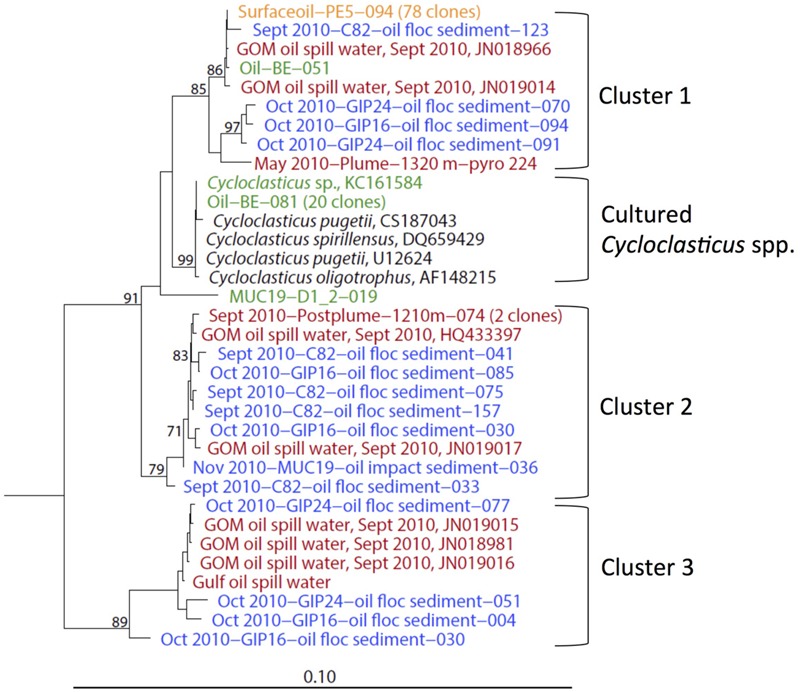
**16S rRNA gene phylogenetic tree of *Cycloclasticus* sequences derived from both water column and the surficial sediment of the Gulf of Mexico at various time points.** The orange-colored clone is from May 2010 surface oil slick; red clones are from post-plume water (Valentine et al., 2010; Redmond and Valentine, 2012; Yang et al., 2016); blue clones are from surficial sediments of September, October and November 2010; green clones represent phylotypes and a new strain from oil slick incubations (Gutierrez et al., 2013) and 4°C enrichments (Yang, 2014).

The obligately polyaromatic hydrocarbon-degrading genus *Cycloclasticus*, previously cultured from Gulf of Mexico sediments amended with phenanthrene or naphthalene (Geiselbrecht et al., 1998), was repeatedly detected in oil polluted surficial sediments from September, October, and November 2010 (**Figure 5**). *Cycloclasticus* was consistently present in surface oil slick collected in May 2010 (Yang et al., 2016), in the deep hydrocarbon plume in June 2010 (Redmond and Valentine, 2012), and in post-plume deep water in September 2010 (Yang et al., 2016). *Cycloclasticus* phylotypes were also enriched from marine snow grown on weathered Macondo oil (Arnosti et al., 2016), in stable-isotope probing enrichments on phenanthrene (Gutierrez et al., 2013), and in sediment incubations with weathered oil slick in the lab (Yang, 2014); a pure-culture isolate from weathered surface oil slick (*Cycloclasticus* sp. TK-8) is now available for detailed study (Gutierrez et al., 2013). These *Cycloclasticus* phylotypes formed phylogenetically tight clusters; three of them consisted exclusively or predominantly of uncultured sequences from the water column and the sediment, and a fourth cluster included the previously described species and the new isolates and enrichments from Deepwater Horizon samples (**Figure 6**). Interestingly, these clusters obtained by full-length 16S rRNA gene sequencing also matched clusters obtained independently by high-resolution oligotyping (Kleindienst et al., 2016). Cluster 2, containing phylotypes from the sediment and water column sampled in September, October and November 2010, corresponded to *Cycloclasticus* ecotypes termed type 02 and 07 that accounted for the majority of *Cycloclasticus* phylotypes in post-plume water column samples (Kleindienst et al., 2016). Cluster 3, which contained phylotypes from sediment and water column sampled in September and October 2010, corresponded to *Cycloclasticus* ecotype 4, a plume population sampled in May and June 2010 (Kleindienst et al., 2016). These mixed seawater and sediment *Cycloclasticus* clusters indicate transportation by sinking particle flux and habitat connectivity: their members followed the continuous sedimentation of oil-derived marine snow, containing oil particles and oil-degrading bacteria, to the seafloor. Since *Cycloclasticus* spp. are obligate aromatic hydrocarbon degraders, the weathered oil-derived aggregates on the seafloor most likely sustained populations of *Cycloclasticus* spp. that had originally arrived on sinking particles. Phylotypes of *Cycloclasticus* persisted into the November 2010 sediments, but were no longer detected in the sediments collected in July 2011 (**Figure 5**).

### Colwellia

Members of the heterotrophic bacterial genus *Colwellia* were continuously detected in deep hydrocarbon plumes and post-plume seawater (Valentine et al., 2010; Redmond and Valentine, 2012; Yang et al., 2016), as well as in seafloor sediments with high concentrations of total hydrocarbon petroleum (Mason et al., 2014); a new *Colwellia* strain was isolated from Gulf of Mexico seawater enrichments supplied with Macondo oil and dispersant Corexit (Baelum et al., 2012). In contrast to *Cycloclasticus, Colwellia* was found in both oily and non-oily sediments in September, October and November 2010. Although the *Colwellia* bloom was initially triggered during the late stage of the deepwater hydrocarbon plume (Valentine et al., 2010), the growth of *Colwellia* spp. does not solely depend on external hydrocarbon supply. This heterotrophic group was previously found in organic-rich sediments ranging from fish farm sediments (Bissett et al., 2006) to Antarctic continental shelf sediment (Bowman and McCuaig, 2003). Since *Colwellia* spp. could be autochthonous to the seafloor surficial sediment, it cannot be unambiguously linked to the oil fallout.

### Bacteroidetes

**FIGURE 7:**
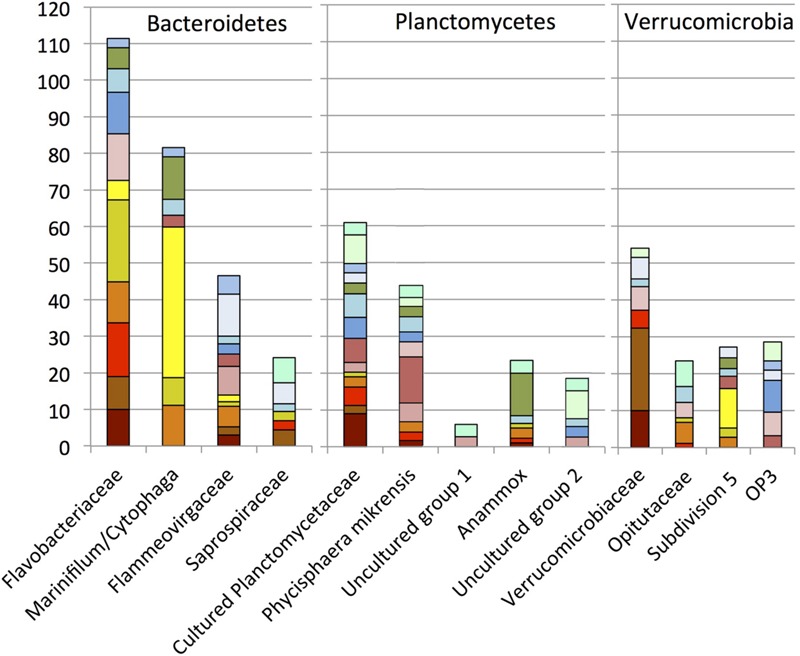
**Order, family- and genus-level identification of 16S rRNA clones representing the phyla *Bacteroidetes, Planctomycetes*, and *Verrucomicrobia* in the sequence dataset shown in Figure 2**.

Two major family level groups, the *Flavobacteriaceae* and the *Marinifilum*/*Cytophaga* group, increased the contribution of the phylum *Bacteroidetes* to the October 2010 oil-impacted samples; the rRNA survey also confirmed their high activity at that time (**Figure 7**). The *Flavobacteriaceae* increased already in the oil-contaminated sediments of September 2010, whereas the *Marinifilum/Cytophaga* group was not detected in the September 2010 sediments and peaked 1 month later in the October oily sediments. Here, the reverse-transcribed 16S rRNA clone library implied high activity of an uncultured *Cytophaga* group; relatives of this group were enriched on crude oil at 5°C, similar to the temperature at the bottom of Gulf of Mexico (Brakstad and Bonaunet, 2006). Several studies indicated that members of the *Flavobacteriaceae* and *Cytophaga* respond to the presence of hydrocarbons. In beach sediments polluted by the 2002 Prestige oil spill in northwestern Spain, members of the *Bacteroidetes* – mostly *Flavobacteria* and *Sphingobacteria* – accounted for up to a quarter of the bacterial clone libraries in samples that were taken 5 years later (Acosta-Gonzalez et al., 2013). Several *Bacteroidetes* isolates are known to participate in the biodegradation of polyaromatic compounds. For example, *Yeosuana aromativorans*, from estuarine sediment and seawater, is capable of degrading PAHs such as pyrene (Kwon et al., 2006; Sheppard et al., 2012), and strains of *Flavobacterium* sp. isolated from sewage were able to grow on biphenyl (Stucki and Alexander, 1987).

### Planctomycetes

Within oil-impacted sediments, the phylum *Planctomycetes* was mostly represented by clades of cultured *Planctomycetes* and by the largely uncultured clade represented by *P. mikurensis* (Fukunaga et al., 2009), originally isolated from algae (**Figure 7**). Many *Planctomycetes* sequences were closely related to those derived from marine seafloor habitats, such as sediments in the South China Sea (Hu et al., 2010), deep sea sediment underlying two whale falls (Goffredi and Orphan, 2010), and seafloor lavas from the East Pacific Rise (Santelli et al., 2008). Their association with marine snow particles (Delong et al., 1993) and the high numbers of sulfatases in marine *Planctomycetes* characterize them as specialists for the initial breakdown of sulfated heteropolysaccharides in marine snow and indicate their importance for recycling carbon from these compounds (Woebken et al., 2007). In the seafloor clone libraries, *Planctomycetes* occur abundantly in individual samples (E01801, E01402) but do not show a preference for oil-impacted sediments; in this regard they contrast with other bacterial groups that are stimulated by fresh oil sedimentation. Clones of the Anammox clade (in marine environments, genus *Scalindua*) were mostly obtained from the GIP3-4 cm sample, indicating a potential enrichment in sediments below the oil-impacted surface layer.

### Verrucomicrobia

Phylotypes of the *Verrucomicrobia* occurred in intermediate abundance in most sediment samples. Our family-and genus-level resolution analysis demonstrated divergent dynamics of the *Verrucomicrobia* subgroups (**Figure 7**). The phylum *Verrucomicrobia* contains a total of seven subdivisions that are not all represented by cultured strains (Hugenholtz et al., 1998; Freitas et al., 2012). The family *Verrucomicrobiaceae*, previously defined as subdivision 1 (Hugenholtz et al., 1998), and containing the methanotrophic genus *Acidomethylosilex* (Freitas et al., 2012), responded to the oil input in early September 2010, whereas members of the uncultured subdivision 5 were appeared only in mid-October 2010 (**Figure 6**). The representative sequences of subdivision 5 were originally cloned from the methanogenic layer of an aquifer contaminated with hydrocarbons and chlorinated solvents (Dojka et al., 1998). Therefore, members of subdivision 5 may be capable of hydrocarbon bioremediation in anoxic environments, consistent with the hypothesis of micro-scale anoxic niches at the seafloor in October 2010.

### PCoA Analysis of Sediment Samples

**FIGURE 8:**
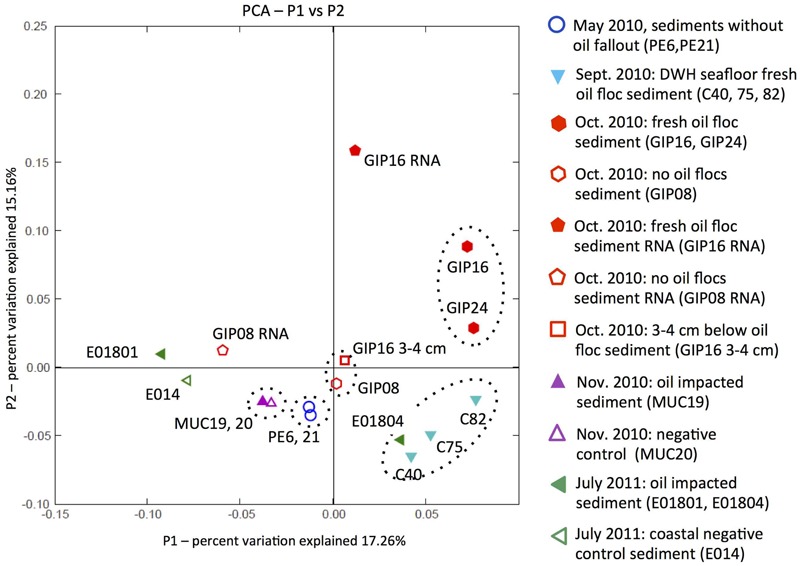
**Principal coordinates analysis (PCoA) of 16S rRNA gene distances between phylotypes in all sediment samples.** Solid symbols stand for the DWH-impacted sediments; open symbols represent the control sediments without visible imprint of oil-derived sedimentation, or samples below the oil-impacted sediment surface, as in the case of GIP16 3–4 cm.

Weighted UniFrac analysis was applied to explore control factors that shaped the clone library structure of the DWH samples using PCoA, based on the phylogenetic affiliations obtained with ARB (Ludwig et al., 2004; Lozupone et al., 2006). Principal components P1 and P2 could explain 32.42% of the variation in bacterial community composition (**Figure 8**). Oil-derived sedimentation was one of the major controls on bacterial community composition, as the principal component P1 clearly separated most oil-impacted samples (P1 value > 0) from the non-impacted control samples. The 2010 September oil floc samples and a 2011 July sample close to the wellhead area (E01804) grouped together, indicating high similarity between these samples. The two contaminated samples GIP16 and GIP24 collected in October 2010 clustered adjacent to, but separately from the September 2010 samples, indicating that their DNA-based bacterial community structures diverged. It is possible to view the September and October clusters as a continuum that could be linked across the two least-distant members of the two clusters, the adjacent September sample C82 and the October sample GIP24 (**Figure 8**). In this view, the bacterial community in the oily sediments is gradually changing over time, potentially in sync with microbially accessible substrate pools or geochemical characteristics of the surficial sediments.

The non-oily samples have P1 and P2 values near or below zero (**Figure 8**). Non-oily samples from May 2010 (PE6 and PE21) and October 2010 (GIP08 and GIP16 3–4 cm), and also the November samples MUC19 and MUC20, formed three clusters that grouped closely to each other, and separately from the oil-impacted September and October samples. The bacterial community of GIP16 3–4 cm shared high similarity with the May 2010 samples, but differed from its oily surface layer (GIP16), consistent with the hypothesis that much of the bacterial community that is usually found in non-oily sediment surface samples persists underneath the layer of oil-derived fallout. Somewhat unexpectedly, the oil-impacted sample MUC19, characterized by the red-brown redox signature of oil-induced metal mobilization (Hastings et al., 2016), groups closely with the distant background sample MUC20. Even more remarkably, this pair is the closest neighbor of the two pre-sedimentation May 2010 samples (**Figure 8**). This result suggests that in late November 2010, specific groups of oil-degrading or oil-responding bacteria no longer determine the overall microbial community composition. The successive enrichments of *Roseobacter, Desulfobacteraceae*/*Desulfobulbaceae, Bacteroidetes*, and *Verrucomicrobia* that structured benthic bacterial communities in oil-impacted samples collected in September and October of 2010 do not last into the late November 2010 samples; instead, the benthic bacterial communities of these samples are reverting to resemble the pre-spill baseline. This does not mean that the oil-derived sedimentation pulse has left no imprint on these samples, but that it is no longer conspicuous on the whole-community level. The July 2011 samples do not show any consistent clustering with each other or other groups of oil- or non-oil impacted sediment samples; it is possible that the microbial communities of these samples from different locations are subject to divergent ecosystem trajectories (seasonal phytoplankton and bacterioplankton blooms; sediment resuspension and oxidative processing; long-term processing of oil-derived substrates) that no longer tie them unambiguously to the DWH oil spill. Interestingly, the 2011 July sample E01804 clusters with the oil floc samples C40, C75, and C82 collected in September 2010, suggesting that close analogs of these oil floc communities may – under specific circumstances – recur in benthic sediments.

The reverse-transcribed RNA samples GIP16 and GIP08 RNA of October 2010 diverge generally from their DNA counterparts, suggesting that the bacterial community patterns of gene expression are different from those based DNA and gene presence. The clustering that is observed on the DNA level might turn out differently if the entire study was replicated on the RNA level.

## Discussion and Outlook

A large proportion of the oil that escaped earlier budgeting efforts (McNutt et al., 2012; Joye et al., 2016) reached the seafloor on the continental slope of the northern Gulf of Mexico by sedimentation of oily marine snow (Passow et al., 2012; Ziervogel et al., 2012), as recently substantiated by comprehensive stable carbon isotopic analyses of seafloor hydrocarbon deposits (Chanton et al., 2015). This deposition event took place in summer and early fall 2010, as dated by ^234^Th decay in surficial sediment cores (Brooks et al., 2015), and it appears to have triggered consecutive microbial population changes that were detected in this clone library survey. In our scenario, the rapid consumption of easily accessible hydrocarbons (short chain alkanes or polar substituted aromatics) or extracellular polymeric substrates in oil-derived marine snow by aerobic heterotrophic bacteria such as *Roseobacter, Verrucomicrobiaceae*, and *Bacteroidetes* in September and October 2010, could lead to localized oxygen depletion close to the sediment-seawater interface, or favor the formation of anoxic zones at micro-scale on the surface of the sediment. Anoxic microniches would provide suitable habitats and substrates for a wide range of anaerobes, such as denitrifying (Mason et al., 2014), metal-reducing (Hastings et al., 2016), or SRB (Kimes et al., 2013) that continue to degrade recalcitrant petroleum hydrocarbons at the seafloor *in situ*. This scenario explains the conspicuous appearance of *Desulfobacteraceae* and *Desulfobulbaceae* in the surficial sediment samples collected in October 2010. The microbial degradation cascade triggered by oil snow deposition on the seafloor seems to have slowed down in late November 2010, in the sense that the overall bacterial community structure is no longer controlled by the oil sedimentation response but resembles again the pre-spill community. The microbial response to petrocarbon deposition remains detectable on finer taxonomic scales when specialized oil-degrading bacterial populations are considered. For example, the obligate polycyclic aromatics degrader *Cycloclasticus* could be traced into the November 2010 sediment sample set.

To link microbial community signatures and hydrocarbon content of the sediment, the literature and databases were searched for chemical analyses performed on the same sediment samples, obtained with the same multicorer deployment. Concentrations of total polyaromatic hydrocarbons (TPAH) and compound-specific results are published for oil-impacted sediment cores collected with R/V Cape Hatteras in Mid-October 2010 near the Macondo wellhead (Woodruff, 2014), including core GIP16 with TPAH concentrations of 2192, 309 and 272 ng/g at 0–1, 1–2, and 2–3 cm depth, respectively, and core GIP24 with TPAH concentrations of 819, 287, and 262 ng/g in 0–1, 1–2, and 2–3 cm depth, respectively. These elevated TPAH concentrations demonstrate the arrival of a sedimentation pulse of petroleum-derived aromatic compounds on the seafloor. At 3 cm sediment depth, the TPAH concentrations approach the average pre-spill TPAH concentrations in sediments of the northern Gulf of Mexico, near 140 ng/g (Wade et al., 2008). Thus, the impact of polyaromatic hydrocarbon availability on microbial community structure should diminish with sediment depth, as indeed observed in the GIP16 3–4 cm sample (**Figure 8**). In general, TPAH concentrations of surficial sediments (0–1 cm layer) sampled in mid-October 2010 around the Macondo wellhead increased to above 400 ng/g, with an average near 1100 ng/g and localized peaks near 17,000 ng/g (Woodruff, 2014). Specific classes of polyaromatic compounds (fluorenes, phenanthrenes, anthracenes, chrysenes, pyrenes) decreased in concentration over the upper three centimeters, consistent with recent deposition from the water column (Woodruff, 2014). On resampling in 2011, TPAH concentrations in surficial sediments had decreased to 200–300 ng/g, with an average of 220 ng/g (Woodruff, 2014); specific polyaromatic compounds had also decreased in concentration by approximately an order of magnitude, presumably reflecting microbial degradation of these substrates (Woodruff, 2014).

As a distinguishing characteristic, the microbial community of the seafloor sediments changed on longer time scales than the oil-impacted community in the water column. The seafloor sediments received the oil-derived sedimentation pulse and its associated microbial communities after the onset of marine oil snow formation in the surface water in early May 2010, and the development of the deepwater hydrocarbon plume in May and June 2010 (Yang et al., 2016). Once the oil-derived marine snow and its embedded hydrocarbon particles started to settle on the seafloor sediment surface, the seafloor acted as an integrator of the microbial and chemical fallout, collecting the oil-derived sedimentation pulse continuously throughout the summer and fall of 2010. The microbial responses of oil-degrading sediment bacteria, or opportunistic heterotrophs that took advantage of other oil snow components, were reflected in conspicuous whole-community changes in September and October 2010. At this time, the bacterial communities in the overlying water column were reverting to pre-spill conditions (Yang et al., 2016) and the prevailing transport direction, toward the west and southwest, moved the oil-impacted water masses and their residual oxygen minima and microbial populations from the study area (Diercks et al., 2010b; Joye et al., 2016). The seafloor sediment integrates the changeable water column input over time, and retains the permanent geochemical archive of the oil-derived sedimentation pulse in the same manner as other events that have impacted the quaternary sedimentation regime in the northern Gulf of Mexico (Ingram et al., 2013). As the increasingly recalcitrant oil-derived fallout is buried by ongoing sedimentation under increasingly anaerobic conditions, slow microbial degradation and assimilation of recalcitrant hydrocarbons should remain detectable with molecular assays and enrichments, for example by ^13^C-analysis of hydrocarbons that are gradually assimilated into the resident bacterial rRNA (Pearson et al., 2008).

## Author Contributions

TY and AT designed the study; TY, AT, LM, and KS collected and recorded sediment samples on research cruises in the Gulf of Mexico. KS also extracted and reverse-transcribed RNA from samples GIP08 and GIP16. BM designed the modified RNA extraction method for marine sediments. SJ served as chief scientist on cruise AT18-02 (November/December 2010), provided additional samples from RV Oceanus cruise in August/September 2010 (OC486) and served as PI on the ECOGIG consortium that subsequently funded TY’s graduate study. TY performed the 16S rRNA gene sequencing, the phylogenetic analyses, and calculated the PCoA plots; AT and TY wrote the manuscript with input from the other authors.

## Conflict of Interest Statement

The authors declare that the research was conducted in the absence of any commercial or financial relationships that could be construed as a potential conflict of interest.

## Footnotes

1 http://blast.ncbi.nlm.nih.gov/

2 https://web.archive.org/web/20090116042509/http://www.xfig.org/
